# Characterization of Innovation to Fight Child Mortality: A Systematic Scoping Review

**DOI:** 10.3389/ijph.2022.1604815

**Published:** 2022-08-15

**Authors:** Bruno Filipe Coelho Da Costa, Bruno Daniel Carneiro, André Ramalho, Alberto Freitas

**Affiliations:** ^1^ Faculty of Medicine, University of Porto, Porto, Portugal; ^2^ Center for Health Technology and Services Research, Faculty of Medicine, University of Porto, Porto, Portugal; ^3^ Department of Community Medicine, Information and Health Decision Sciences, Faculty of Medicine, University of Porto, Porto, Portugal

**Keywords:** scoping review, innovation, child mortality, under-five mortality rate, neonatal mortality rate, sustainable development goal

## Abstract

**Objectives:** This study aims to summarize how child mortality—a Sustainable Development Goal stated by the United Nations—has been explicitly addressed in the context of innovations.

**Methods:** A scoping review following the PRISMA-ScR Statement was performed analysing indexed and non-indexed literature.

**Results:** Empirical and non-disruptive innovation in the context of process targeting under-five mortality rate was the main subset of literature included in this article. The increment of literature on innovation in the context of SDGs over the last years denotes its growing importance and even though innovation aiming to reduce child mortality is currently being done, a significant part of it is not published in indexed databases but as grey literature.

**Conclusion:** Empirical, disruptive innovation under a structural approach and empirical, non-disruptive innovation under a project point of view are the main types of innovation addressed in the literature and would be of utmost potential to reduce child mortality rate. A systematic review of the methods used for the measures of evaluation of applied innovations, their quality and results would be of great importance in the future.

## Introduction

The Sustainable Development Goals (SDGs), developed by the United Nations in September 2015 [1], are a set of 17 goals with 169 targets over a wide spectrum of the human condition [2]. The SDG 3 directly addresses health, pointing out as its major goal to “Ensure healthy lives and promote well-being for all at all ages” [3]. Child mortality is stated by the United Nations as a Sustainable Development Goal (SDG 3.2) reflected by two indicators. The under-five mortality rate is defined as the probability of a child born in a specific year or period dying before reaching the age of 5 years, expressed per 1,000 live births [4], and the neonatal mortality rate is defined as the probability of a child born in a specific year or period dying during the first 28 completed days of life, expressed per 1,000 live births [5]. SDG3.2 major purpose is to end preventable deaths of newborns and children under 5 years of age, with all countries aiming to reduce neonatal mortality to at least as low as 12 per 1,000 live births, and under-five mortality to at least as low as 25 per 1,000 live births by 2030 [6]. The decline in child mortality over the past 2 decades has been described as one of the greatest successful stories in global health [7]. However, the progress made in reducing the annual global number of under-five childhood deaths from 9·8 million in 2000 to 5·9 million in 2015 [8] is not satisfactory to meet the United Nations’ Sustainable Development Goal targets, and it is estimated that in 2030, 4·4 million children will still die before they reach the age of five [8], if current downward trends maintain the same. Particularizing, Somalia is one of the countries with the highest mortality rates among children aged 5 or younger [9, 10]. The prolonged armed conflict this country has faced as well as an underfunded health system with rudimental childbirth conditions, precarious nutritional conditions, and a low level of vaccination [10, 11] are the main determinants of the extremely high under-5 mortality rate of 115 per 1,000 live births in 2020 (considering the under-5 mortality rate of 66 per 1,000 live births in 2020 of low-income countries in general) [12]. Botswana is another Sub-Saharan country where access to appropriate healthcare for children remains a challenge, as evidenced by the high under-five mortality rate and integrated management of childhood illness indicators [13]. Besides, the COVID-19 pandemic has worsened the already fragile situation and SDG 3 is pointed out as one of the most affected SDGs in the wake of the COVID-19 crisis [14], emphasizing the current global need to point efforts out on this matter. Although several countries and entities have been developing initiatives, the progress towards the achievement of the Sustainable Development Goal of reducing under-five mortality is lagging in many countries, particularly in Africa [15] (as the example of Somalia and Botswana showed) and governments have a key leadership role to play in this process [16] relying on four “imperatives”: innovation, sustainability, measuring outcomes, and mutual accountability [17, 18]. Focusing on innovation, literature states that innovation is a way of well succeeded implementation of the integrated management of childhood, drastically reducing child mortality [13]and its need has become critical to enhancing the quality of care [19]. Innovation may be described as the intentional introduction and application within a role, group, or organization, of new ideas, processes, products, or procedures, to the relevant unit of adoption, designed to significantly benefit the individual, the group, or wider society [20]. Innovations may be taken as a range from evolutionary to revolutionary [21–24]. Evolutionary innovations are those that update the performance of already existing ideas, concepts, products, models or services, with an orientation toward today’s customers/users. On the other hand, revolutionary innovations represent the sustenance of creation, entrepreneurship, and development, focused on the orientation of tomorrow’s customers/users. They are the basis of an enormous number of new technologies, services, or products. Organizations, in general, require both revolutions and evolutions to operate sustainably and profitably in the long term [25]. Based on these definitions, another kind of innovation classification is also often used. Reflecting its impact on stakeholders, innovation can be categorized as either non-disruptive or disruptive [19]. A non-disruptive innovation may be seen as an evolutionary type of innovation. Its focus is to improve something already created (in terms of lowering costs, improving usability, effectiveness), but never compromises its continuous updating [26]. In general, non-disruptive innovation is linked to quality improvement and usually requires less risk from the responsible entity or organization. Instead, revolutionary innovations are more frequently called disruptive innovations. Other possible names for it could be transformational, non-linear, and radical [26, 27]. In this case, a totally new perspective is introduced by this kind of innovation, which creates new concepts, ideas, or products, and usually has a major impact on the stakeholders who implement them. Besides, health care innovations may be classified according to their focus on product, process, or structure [19]. “Product” refers to a materialized thing, a good, or a service. An example of product innovation is the creation of a new solar-powered incubator for infants. On the other hand, process innovation denotes something new related to the production or delivery method. As Varkey et al. mentioned, “the process is required to deliver a product or service and to manage the relationship with the various stakeholders.” A process innovation is, for instance, telemedicine: the principles of medicine practice are the same, but they are presented to the health care provider in a different way. In terms of what refers to structural innovations, they are linked to the infrastructure. This means they embody alterations in the way health care is delivered [19]. As an example, we may mention the medical practice transformation, where physicians quit working only by themselves, going to peoples’ houses, to work in integrated teams at hospitals [28]. The most important components of innovation are creativity, first implementation, and recognition of value [19]. This concept deeply depends on information, knowledge flow, and networking in such a way that low levels of collaboration and interactions among the main actors of innovation are one of the principal factors hampering innovation deployment [19]. As a result, governments are not the only institutions that are implied in this topic. The Triple Helix model, which brings together the entities university, industry, and government, enables international cooperation in accelerating the process of transferring scientific and technological knowledge [29], making health innovation critical.

Assuming the late interest and demand for this theme, the main objective of this study is to summarize how child mortality has been explicitly addressed in the context of innovation, providing an opportunity to map key concepts, gaps in evidence, and research. In addition, we take the opportunity to explore how this health indicator is discussed as a sustainable development goal (SDG). Additionally, we investigated the innovations retrieved to address the child mortality target as an indicator of the health system’s quality and to what extent they are considered in their approaches as an objective of sustainable development.

## Methods

**FIGURE 1:**
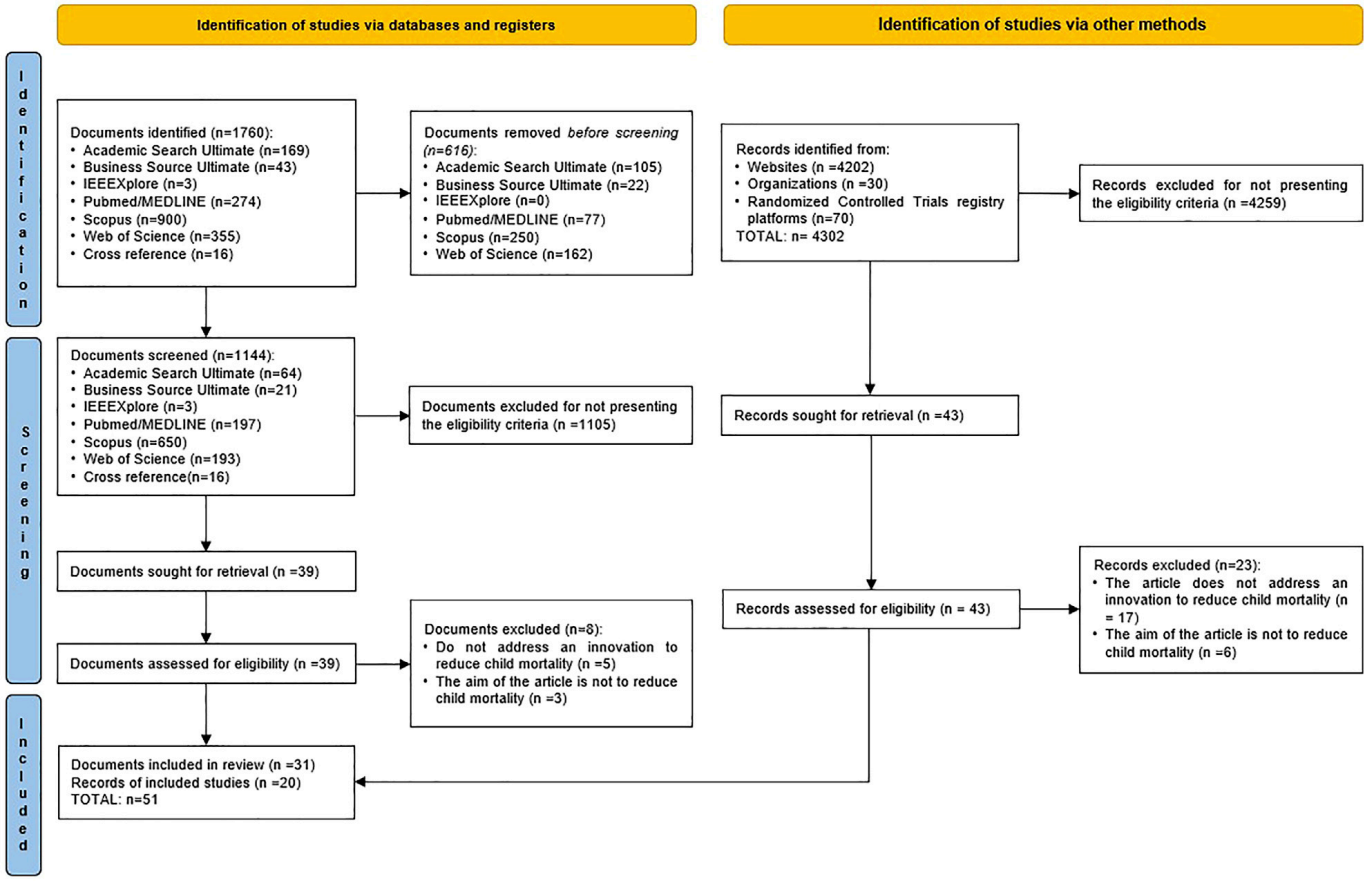
Study selection flow diagram, based on The Preferred Reporting Items for Systematic reviews and Meta-Analyses 2020 statement: an updated guideline for reporting systematic reviews (Australia, 2021).

This scoping review was conducted following the PRISMA Extension for Scoping Reviews (PRISMA-ScR) [30, 31], thus employing a systematic approach to identifying evidence on innovations to fight child mortality (Figure 1). The protocol was developed by all the authors. We follow the PROSPERO database protocol model to ensure study transparency and reproducibility, although scope reviews are not eligible for registration [32, 33].

### Search Strategy

The search expression was defined according to the main purposes of this study. It assembles two domains: child mortality and innovation. An initial search term was defined based on the terms found in published articles by the authors’ previously known subjects, MeSH and related terms, synonyms, and others found in relevant articles and documents from the World Health Organization and the United Nations. The query was then calibrated through a sensitivity analysis of these terms [34], which included two rounds. The study was conducted from inception to 26th October 2021 and included searches in the following six indexed-literature databases: Scopus, Medline/Pubmed, Web of Science, IEEEXplore, Academic Search Ultimate, and Business Source Ultimate. Complete search expressions used in each database are presented in Supplementary Material S1. Regarding grey (non-indexed) literature, a free, snowball-based search was performed, tracking general sources of this type of literature (e.g., OpenGray, Carrot2, Millionshort, Google Search), specific sources (including organizations’ websites, international reports, publications, and databases directly related to the topic of this review) and clinical trials registry platforms. The list of the grey literature consulted can be found in the Supplementary Material of this article as well (Supplementary Material S2).

Language and time constraints were not considered when conducting the search.

### Selection Criteria

The included studies fulfilled the following criteria:

(1) Studies addressing child mortality.
(2) Studies addressing innovation to reduce child mortality.
(3) Peer-reviewed, indexed, and non-indexed literature.

Studies were excluded for the subsequent motives:

(1) Publications not meeting inclusion criteria.
(2) Editorials, short surveys, letters, narrative reviews, and experts’ opinions.
(3) Literature whose abstract was not available in the screening phase or which, in the eligibility phase, did not have the full-text version available (even after direct contact with the authors).

### Screening Phase

Once we obtained all the articles, duplicates between databases were identified and excluded using the bibliographic manager software Endnote X9 (The EndNote Team, Clarivate, 2013, Philadelphia, United States of America). From 1760 articles, a total of 1,144 remained and were evaluated in the screening phase (reading of title and abstracts) by two independent reviewers. The resolution of divergences was achieved by consensus. In this study, Cohen’s kappa coefficient (κ) was used to measure inter-rater reliability for qualitative items, in the case of the decision to “INCLUDE” or to “EXCLUDE” each literature document retrieved (k = 0.618; 95% CI = 0.49–0.75). According to Kraemer (2014), it is a more robust statistical measure of agreement, in comparison to the simple percentage calculation of agreement. By evaluating the reproducibility process, we can ensure the transparency of the applied methods, permitting a user to address the same question and screen the same set of literature to come up with a comparable general conclusion. Concerning grey literature, 4,302 retrieved documents were evaluated by the same two reviewers in the screening phase. Once more, a consensus method was selected to achieve resolution of divergences.

### Eligibility Phase

After the screening phase, full texts of all the included indexed articles were extracted (*n* = 39). As it was planned to contact the corresponding author if the full text of the article was not available, we used the ResearchGate website to extract the available attached full-text articles. The eligible documents were assessed in full-text format. The eligibility criteria were reapplied by two independent reviewers. Additionally, the authors checked the reference lists of each eligible study with the purpose of searching for any omitted literature in the database search. Once more, all the divergences were solved by consensus meetings with the advisory board as tiebreakers, ensuring the eligibility criteria were clear and rigorously applied, and a kappa statistic was calculated to ensure inter-rater reliability (k = 0.53; 95% CI = 0.193–0.868). The eligibility criteria were also applied by two independent reviewers concerning grey literature and, after reaching a consensus, all the documents considered to fulfil eligibility criteria were included.

### Charting the Data, Summarizing, and Reporting the Findings of Included Literature

A standard data extraction form was created by the authors, and general data was extracted from each study. For both indexed and non-indexed literature, we recorded information on the first author, year of publication, country of publication, source of data, definition of child mortality addressed in the study, concept of research, content of research, context of research, description of innovation to reduce child mortality, collaborations addressed in the study, and main findings of the study. The classification of innovation used in this scoping review in terms of concept, content, and research context was based on previous literature [19, 35].

## Results

This Scoping Review was performed by searching for evidence-based on two approaches: indexed literature and non-indexed literature (Figure 1). The database searches conducted in October 2021 retrieved 1760 potentially relevant indexed studies. After duplicate removal, 1,144 articles were screened. Following title and abstract screening, 39 full-text articles were selected for eligibility criteria assessment, and 31 articles were included for analysis. At inception, 4,302 potentially relevant non-indexed records were found, of which 4,259 were excluded for not meeting the eligibility criteria. From the 43 records that went through the eligibility phase, 20 were included for analysis. Thus, a final total of 51 documents were included from both indexed and non-indexed sources and were analysed.

### Studies Characteristics

**FIGURE 2:**
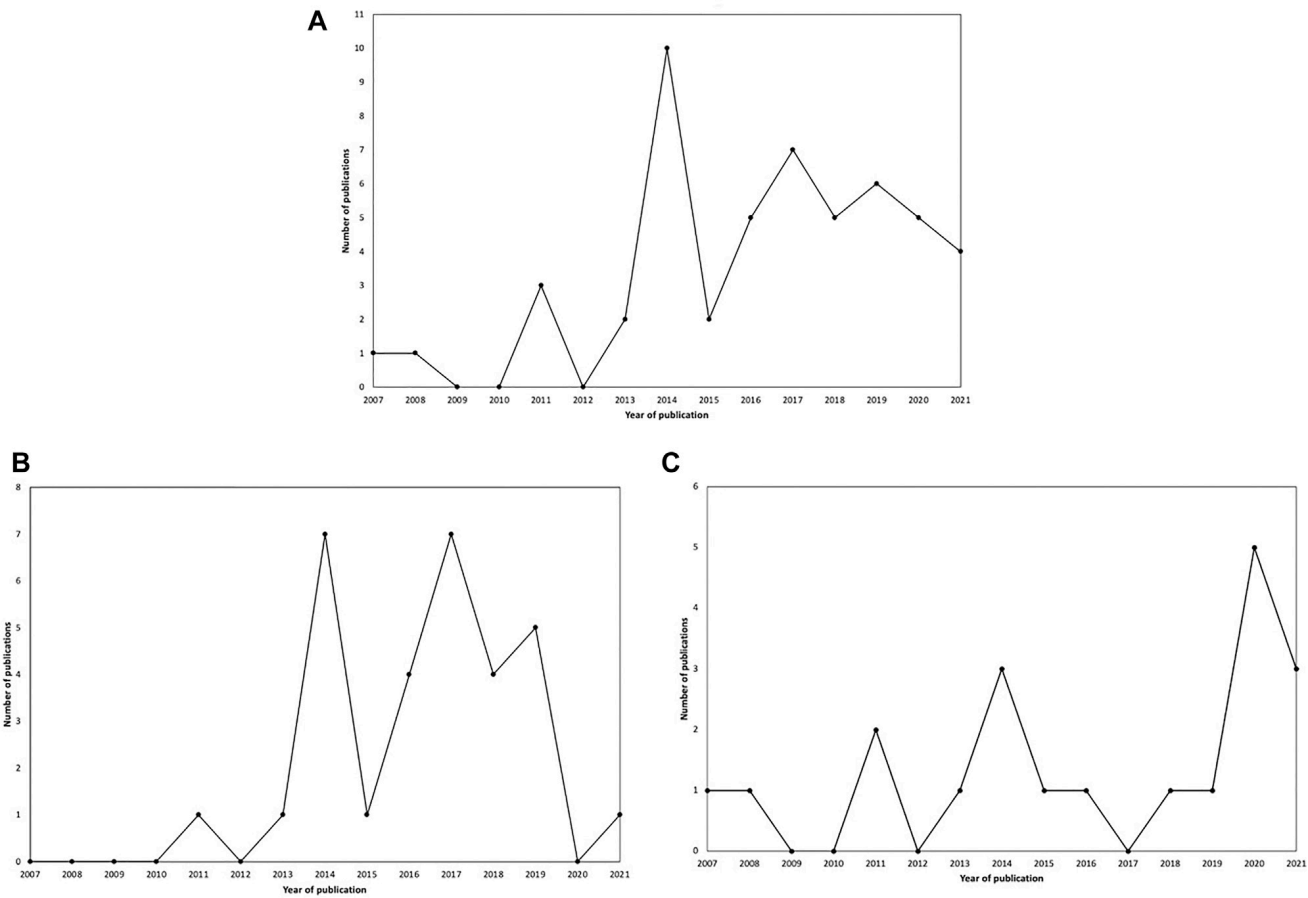
Number of publications per year and type of data source regarding retrieved documents. **(A)** Indexed and non-indexed literature; **(B)** Indexed literature; **(C)** Nonindexed literature (Portugal, 2021).

The main characteristics extracted from the included studies can be seen in Supplementary Table S1. Two publication peaks occurred over time (Figure 2) first being in 2014 (*n* = 10) for both data sources. The second was remarkable in 2017 and extended to the present day (*n* = 28 so far), but with few documents on grey literature, despite the noteworthy increase in the number of publications in 2020 for this source type. Regarding the analysed documents’ country of origin, it reveals that the USA was the most frequent publisher, counting 19 publications. India followed with four publications. Brazil and Ireland counted three publications each. Kenya, Switzerland, and Peru each published two documents, while New Zealand, Uganda, Columbia, the Philippines, the Netherlands, Senegal, Korea, Malaysia, Denmark, Cameroon, Canada, Portugal, France, Ghana, Armenia, and Zambia each published one. The under-five mortality rate was the most addressed child mortality indicator, namely in 39 studies (76%), and the neonatal mortality rate was mentioned in 23 of the analysed publications. There were eight publications [36–43] with a conceptual approach to innovation addressing child mortality. From the empirical point of view (*n* = 43), it is perceptible that most of the evidence focused on a longitudinal method of innovation application. The second most frequent empirical method was in the laboratory research context. On the other hand, a disruptive innovation standpoint was present in nine of the documents [41, 44–51]. It is possible to notice that, of these 9 disruptive innovations, most of them were found in non-indexed sources (*n* = 5). Besides, disruptive innovation is mostly related to the context of structural innovation. The process perspective was the most common when it came to the context of research, since it was discussed in 26 of the documents (51%). Instead, product creation/development innovation was the least prevalent in the included literature (*n* = 11). It’s worth noting that in each document, just one sort of innovation context has been applied. From the perspective of entities involved in the creation/development of each presented innovation and/or entities for whom these innovations may be directed, “academy” was individually addressed in 14 studies, followed by “policy makers” (*n* = 12). Shift Labs, Inc., 2018 and M-Chanjo Association, 2014 presented innovations exclusively regarding “companies”. Two of the included documents approached both “academy and companies” perspectives [37, 52] and two others approached both companies and policymakers [53, 54]. Sixteen studies addressed “academy and policy makers” interfaces. Concern Worldwide, 2014; Concern Worldwide, 2016 and Universidad Peruana Cayetano Heredia, 2020 were the only studies addressing all the mentioned entities. It is worth mentioning that most of the included literature is framed into qualitative analyses of proposed innovations rather than presenting quantitative measures.

Innovation combining an empirical and non-disruptive approach was the most present in literature (*n* = 34), focusing on the context of process (*n* = 21) and followed by product (*n* = 10) and Structural (*n* = 3). On the other hand, conceptual and disruptive innovation was only targeted by the United Nations Organization, in 2013, when it proposed “GAPPD—The integrated Global Action Plan for the Prevention and Control of Pneumonia and Diarrhea”. In this Scoping Review, Empirical and Disruptive innovation totalized 9 publications and Conceptual and non-disruptive innovation were covered in 7 documents. In the first case, the most frequent context of innovation was structural, with a total of 8 publications. Process was the most lectured context in terms of conceptual and non-disruptive innovation (*n* = 4). The number of studies for content and concept of innovation by context was summarized and shown in Table 1.

**TABLE 1:** Number of studies for innovation concept and content by context (Portugal, 2021).

Concept and content	Context			Total
	Process (n)	Product (n)	Structural (n)	
Empirical, Disruptive innovation	1	0	8	9
Empirical, Non-disruptive innovation	21	10	3	34
Conceptual, Disruptive innovation	0	0	1	1
Conceptual, Non-disruptive innovation	4	1	2	7
Total	26	11	14	51

As previously mentioned, under-five mortality rate is the most mentioned child mortality indicator in the selected studies. The main subset of literature included in this article (*n* = 17) was empirical and non-disruptive innovation in the context of a process aimed at lowering the under-five mortality rate. Neither the neonatal mortality nor the under-five mortality rates were probed by the following innovation methods: conceptual and disruptive innovation in the context of process; empirical and disruptive innovation in the context of product; conceptual and disruptive innovation in the context of product. From the studies aiming both neonatal mortality rate and under-five mortality rate indicators (Articles ID: 4; 7; 10; 13; 29; 33; 37; 39; 41; 45; 46), most of them fit into a structural context (Articles ID: 4; 7; 13; 33; 45; 46). Table 2 shows the innovation methods by child mortality indicators (SDG goals).

**TABLE 2:** Innovation methods by child mortality indicators (Portugal, 2021).

Innovation methods by context	Neonatal mortality indicator^a^	Under-five mortality indicator^a^
Process		
Empirical, Disruptive innovation	NA	32
Empirical, Non-disruptive innovation	11, 19, 21, 23, 29, 39	1, 5, 15, 16, 22, 25, 29, 30, 31, 35, 38, 39, 40, 42, 43, 44, 51
Conceptual, Disruptive innovation	NA	NA
Conceptual, Non-disruptive innovation	36	18, 28, 50
Product		
Empirical, Disruptive innovation	NA	NA
Empirical, Non-disruptive innovation	6, 10, 12, 37, 41, 47	8, 10, 27, 34, 37, 41, 48
Conceptual, Disruptive innovation	NA	NA
Conceptual, Non-disruptive innovation	9	NA
Structural		
Empirical, Disruptive innovation	4, 14, 33, 45, 46	2, 4, 20, 26, 33, 45, 46
Empirical, Non-disruptive innovation	17	24, 49
Conceptual, Disruptive innovation	7	7
Conceptual, Non-disruptive innovation	3, 13	13

a Included studies ID. NA, not addressed.

## Discussion

As far as we are concerned, there are no scoping reviews addressing innovation aiming to reduce child mortality. The increment of literature on this subject in the context of SDGs over the last few years indicates its growing importance. The peak of publications verified in 2014, a year before the transition from Millennium Development Goals to Sustainable Development Goals [55], suggests that at this time, countries paid more attention to innovation in the context of child mortality in order to achieve the established SDGs. Regarding documents’ country of origin, it is interesting to notice that the major publisher is the USA, a country that already accomplished SDG 3.2 with an under-five mortality rate of 6.7 deaths per 1,000 live births (IC95% = 6.1–7.4) and a neonatal mortality rate of 3.9 deaths per 1,000 live births (IC95% = 3.5–4.2), in 2017 [56]. However, the USA has poorer children’s health outcomes than other wealthy nations despite greater per capita spending on health care for children [57] and, comparing to other high-income nations, the USA presents higher mortality rates as well as lower life expectancy at birth [58, 59], possibly suggesting the country´s concern about this area. On the other hand, a longitudinal method to innovation application was the most frequent in the literature analysed in this article, meaning the study idea was applied (or is still under application) to a population over a period. As a result, this could be a bad omen for these innovations, given that some literature claims that several promising technological innovations in health and social care are not sustained for a long time at the organizational or system-level [60]. The fact that only a small number of collected documents regard disruptive innovation is according to existing literature, which mentions a greater difficulty in its implementation, either because of questions related to incentives and an economical point of view or because of its adoption and diffusion issues [61]. Besides, it is essential for everyone who wants to learn more about disruptive innovation in the context of child mortality to search not only for literature in indexed databases but also in non-indexed sources once most of the disruptive material was found in the grey literature. Innovation aiming to reduce child mortality is being done, but currently a significant part of it is not published in indexed databases but as grey literature. Although the included articles mainly presented a single type of innovation context, we must comprehend that the most successful innovators admit that integration and conjugation of innovation types is vital; in particular, a product innovation not complemented at any level by a process or system innovation may have difficulties in its approval and application [19]. Furthermore, one of the reasons for the innovation gap in the Mediterranean area, compared to the main global competitors, is the limited capacity to bring the knowledge generated into the market by creating a stable and strong link between research (academies) and business (companies) [29]. Only two studies establishing a link between academia and businesses were found during our scoping review, proving this point.

Disruptive innovation is a threat to the value of previously existing services and organizations offering them [62]. As a result, top provider organizations that produce these types of breakthroughs but whose large revenues are threatened by these same technologies, seem to impede or even obstruct their adoption [62]. This corroborates our results, considering the vast amount of analysed literature was related to non-disruptive innovations. Thus, it is perceptible that the great quantity of retrieved disruptive innovations (10 articles) is associated with a structural context (9 articles), which is also supported by precedent literature, mentioning that structural innovations, usually affecting the internal and external infrastructure and creating new models, are more likely to present as disruptive; they represent major changes in the way healthcare is delivered [19]. Analysing the context of innovation addressed by recovered documents, a process innovation was first lectured, approving the fact that process enhancements may be lower in cost but allow for an earlier return of value [19] and thus be more advantageous. Product innovations can produce substantial near-term publicity, but their value is usually realized farther in the future, usually demanding years of investment, particularly due to regulatory requirements [19], explaining their smaller appearance in literature. Although the under-five mortality rate was the most common indicator of child mortality in the literature, we cannot assume that the neonatal mortality rate received less attention. We must be aware that some literature chooses to subdivide under-five mortality rates into age groups, with the neonatal mortality rate being diluted or included in these groups, and therefore, not being mentioned as the neonatal mortality rate [63]. By performing this scoping review, we also understood that even though child mortality is an SDG target, some literature addresses its indicators (under-five mortality rate and/or neonatal mortality rate) without discussing them as a way to achieve these SDGs. According to our results, the scientific community should be aware that child mortality is a keystone for sustainable human development and innovation is essential in this context. Thus, the responsibility for developing the evidence base supporting connected care is shared between organizations funding health services research, investigators, provider organizations, individual providers, payers [62], companies, governments, and policy-makers. All of them must not only seek knowledge and innovation but also exchange them, promoting their evolution, diffusion, and application. One of the great challenges remains to ensure widespread availability of these useful services [62] and newly proposed ideas. The disparities and inequities between countries at resource and infrastructural levels, and the nonexistence of a planning framework in some cases, represent a threat to countries’ attempts to make substantial progress in child mortality. It is important to remember the need for guiding and coordinating funds and investments on this topic as well as strengthening health systems so as to achieve the 3.2 target for SDGs. Furthermore, we must remember that the role of primary health care is crucial to reducing child mortality, and a variety of innovations can help in timely decision-making to prevent this unfavourable outcome.

### Study Limitations

Despite the consistency of the retrieved information, our study has some limitations. By covering a broad range of literature, indexed and non-indexed, without time or language restrictions, we managed to present a robust and representative perspective on the studied topic. Besides, by exploring databases related to diverse main areas (such as biomedical sciences, technology, business, economy, scholar-based and multidisciplinary) along with exploring and presenting cross-references, we maximized the assessed literature. On the other hand, its main limitation is related to the fact that new products, processes, or structural concepts that have not been explicitly classified as innovations in literature may not have been retrieved. However, given the vast amount of published material in the field, not restricting the search expression to terms associated with innovation and instead looking at all literature under the single domain of child mortality would be impractical. The option of assessing non-indexed literature sought to minimize this limitation. Despite the robustness of our results, we suggest that new studies to capture existing innovations, especially those that have not been published anywhere, should be aggregated in the future. Considering most of the articles included in our study adopted a qualitative rather than a quantitative approach to provide analysis regarding innovation aiming to reduce child mortality, the heterogeneity of the methods described, and the missing outcome measures, it would not be easy to perform a meta-analysis on this subject. Employing quantitative methods to conduct more data-driven research and using real data to provide an adequate and reliable assessment of innovation [14] in the context of child mortality is highly recommended for future research.

### Future Directions and Conclusion

Our scoping review demonstrates interesting aspects of innovation related to child mortality and proves to be pertinent in a relatively difficult field to innovate in. Increasing the availability of data, from surveys to censuses to facility records, would greatly strengthen the precision of local monitoring of child health needs [63], allowing the scientific community, investors, and policymakers to understand which areas should be prioritized for creating, developing, and applying methods and assessment measures aiming to reduce child mortality. Recovering the examples of Somalia and Botswana, most of the included literature could be used as a strong weapon to decrease child mortality. The widespread of the “Doula program” in these low-income countries is one of the possibilities, by increasing the provided perinatal services (community-based childbirth education classes, labour and delivery support, postpartum mom/baby care and instruction focusing on mom/baby attachment, and extension of breastfeeding duration). Also, even though mobile phone penetration as a percentage of population reached less than 50% in 2019 in Somalia according to World Bank [64], Milktrack mobile application would help reduce infant mortality mostly by preventing undernutrition and stunted growth among children by empowering the breastfeeding practice. Therefore, the implementation and reinforcement of telecommunication networks and mobile phones’ access would be of extreme importance to Somali people allowing the use of this type of innovation. Following the same line of thought, another innovation of great interest could be dissemination of M-Chanjo app. The usage of this mobile Health platform would help disseminate information on childhood vaccines, through text message alerts. As previously mentioned, low levels of vaccination in Somalia are one of the main reasons why child mortality is so high in this Sub-Saharan African country, justifying its importance. “Doula program,” Milktrack mobile application and M-Chanjo app represent the most common type of innovations, as concluded in this scoping review. The first one regards empirical, disruptive innovation under a structural approach and the others respect empirical, non-disruptive innovation from a project point of view. Then, these could be pointed out as the type of innovations with the greatest potential to be applied aiming to reduce child mortality rate.

To finish, this study offers a contextual outline that might be valuable for driving the construction of future studies as well as some clues for future policies in the context of the SDGs, namely child mortality. A concrete suggestion for future research could be a more detailed and advanced systematic review of the methods used for the measures of evaluation of applied innovations and their quality. It would allow us to get more precise measures about the application of a concrete innovation in a population, letting us objectively understand whether that innovation is useful or not to achieve the target of reducing child mortality worldwide. On what concerns to innovations considered time-consuming and of limited added value yet feasible, interesting, novel, ethical, and relevant, further pilot studies could be made evaluating those innovations’ performance on specific populations of potential interest. On the other hand, regarding innovations seen as useful tools to globally reduce child mortality, larger trials validating these findings could be made, providing high-quality evidence to inform policy decision-making. It is important to emphasize that additional efforts are required as current trends do rule the world out of the achievement of SDGs for reducing child mortality.

## Abbreviations

ARIDA, Acute Respiratory Infection Diagnostic Aid; CoIIN, The Collaborative Improvement & Innovation Network; CRISPR, clustered regularly interspaced short palindromic repeats; ECG, electrocardiogram; EHC, Enhanced Health Care System; GAPPD, The integrated Global Action Plan for the Prevention and Control of Pneumonia and Diarrhea; GISSA, Intelligent Governance Framework for Brazilian Health System; HBB, helping babies breathe; IV, intravenous; KMC, Kangaroo Mother Care; LAIS, Intelligent Health System Analyzer; LED, light emitting diode; LiST, Lives Saved Tool; ML, machine learning; NRHM, The National Rural Health Mission; OHW, one heart world-wide; PRISMA-ScR, PRISMA Extension for Scoping Reviews; RFID, radio-frequency identification; SDG, sustainable development goal; SPRINT, Scaling Pneumonia Response INnovaTions; Supporting LIFEeCCM App, Supporting Low-cost Intervention For disEase control and Community Case Management App; UNICEF, The United Nations International Children’s Emergency Fund; VMAPs, Vaccine MicroArray Patches; WebRTC, Web Real-Time Communication; WoT, Web of Things.
