# Supplementary material for: Characterization of Innovation to Fight Child Mortality: A Systematic Scoping Review
Source: Int J Public Health. 2022 Aug 15;67:1604815. doi: 10.3389/ijph.2022.1604815 (PMC9421644; doi:10.3389/ijph.2022.1604815)
Supplement: Supplementary file 1 [file Table1.docx]

**Table S1.** Characteristics of the included studies

| **Article ID** | | **Author** | | **Year** | | | | **Country** | | | | | **Source of data** | | | | | **Definition of Child Mortality** | | | | **Concept of research** | | | | **Content of research** | | | | **Context of research** | | **Description of innovation to reduce child mortality** | **Collaborations (Academy-Companies-Policymakers)** | **Main findings** |
| --- | --- | --- | --- | --- | --- | --- | --- | --- | --- | --- | --- | --- | --- | --- | --- | --- | --- | --- | --- | --- | --- | --- | --- | --- | --- | --- | --- | --- | --- | --- | --- | --- | --- | --- |
| 1 | | Population Council | | 2007 | | | | USA | | | | | Non-indexed literature | | | | | Under-five mortality rate | | | | Empirical, longitudinal research | | | | Non-disruptive innovation | | | | Process | | An innovative program—employing nurses on motorbikes and using community volunteers—attends to deliver health care to people in their own homes | Policy makers | The program has succeeded in cutting deaths among children younger than five years by more than half and is on track to achieve a two-thirds reduction in the next few years. The program has also increased contraceptive use, reduced fertility, and sought to reduce or eliminate female genital mutilation/cutting |
| 2 | | Health Resources and Services Administration of the USA | | 2008 | | | | USA | | | | | Non-indexed literature | | | | | Under-five mortality rate | | | | Empirical, longitudinal research | | | | Disruptive innovation | | | | Structural | | The "Doula program" provides free perinatal services to low-income pregnant women that are at risk for poor birth outcomes. It includes community-based childbirth education classes, labour and delivery support, postpartum mom/baby care and instruction focusing on mom/baby attachment, extension of breastfeeding duration and interconception care | Policy makers | Health Resources and Services Administration of the USA |
| 3 | | Denktas, S. *et al* | | 2011 | | | | The Netherlands | | | | | Indexed literature | | | | | Neonatal mortality rate | | | | Conceptual | | | | Non-disruptive innovation | | | | Structural | | "Ready for a Baby" program settles on measures that consider risk guided care, orientation towards shared-care and improvement of collaborations between health care professionals | Academy AND Policy makers | The first findings suggest that the best results will be achieved from a local approach that is tuned to the local needs, concentrating on the deprived urban areas, in combination with city-wide policies customised to the characteristics of the city itself |
| 4 | | Social Development and Health Secretariat of Sobral | | 2011 | | | | Brazil | | | | | Non-indexed literature | | | | | Under-five mortality rate; Neonatal mortality rate | | | | Empirical, longitudinal research | | | | Disruptive innovation | | | | Structural | | "Four leaf clover" is a program that works on three basic lines of action (care management, surveillance of maternal and child morbidity and mortality and social support), taking into account process and result indicators that complement information systems. This provides feedback for the actions of the Centres and the Program | Policy makers | Since its implementation (2001) until 2010, the program reduced Infant mortality rate at 18.5 in 2009 (per 1,000 live births), in Ceará State, Brazil. Perinatal mortality rate decreased since 2002, from 28.4 to 16.5 per 1,000 live births in 2007. It has a lower cost average than traditional models and, in addition, reduces hospitalizations |
| 5 | | Pesinet Organisation | | 2011 | | | | France | | | | | Non-indexed literature | | | | | Under-five mortality rate | | | | Empirical, longitudinal research | | | | Non-disruptive innovation | | | | Process | | "Pesinet Child Mortality Pilot program" is an approach where healthcare agents carry mobile phones on their visit to family´s houses. They look for five key symptoms (fever, vomiting, diarrhoea, coughs, and weight loss), record the data on a Java mobile application, send the data to a doctor at the local health care centre. Once the doctor receives the agents' data, immediate feedback and recommendations are provided. | Policy makers | 96% of the mothers enrolled in the program reported being satisfied with the service, and 99% said they would recommend Pesinet to friends and family |
| 6 | | The World Health Organization Odon Device Research Group | | 2013 | | | | Switzerland | | | | | Indexed literature | | | | | Neonatal mortality rate | | | | Empirical, longitudinal research | | | | Non-disruptive innovation | | | | Product | | "Odón device" is a low-cost technological innovation to facilitate operative vaginal delivery. It was designed to minimize trauma to the mother and baby | Academy AND Policy makers | Should this method prove to be effective and safe, it could contribute to reduce maternal and newborn complications related to childbirth (such as mortality), particularly in the most vulnerable populations with the least access to quality emergency obstetric care |
| 7 | | United Nations Organisation | | 2013 | | | | USA | | | | | Non-indexed literature | | | | | Under-five mortality rate; Neonatal mortality rate | | | | Conceptual | | | | Disruptive innovation | | | | Structural | | "GAPPD - The integrated Global Action Plan for the Prevention and Control of Pneumonia and Diarrhea" is a plan that proposes an innovative approach for integrating the planning, delivery and monitoring of health interventions for the above mentioned diseases | Academy AND Policy makers | Countries will need to establish mechanisms to ensure that data collected by community health and similar workers is incorporated into reporting systems which will allow the monitoring of GAPPD target indicators |
| 8 | | Hall, V. *et al* | | 2014 | | | | USA | | | | | Indexed literature | | | | | Under-five mortality rate | | | | Empirical, focus group | | | | Non-disruptive innovation | | | | Product | | "The IncuLight: Solar-Powered Infant Incubator" is a portable, readily powered, low cost incubator | Academy | This device presents a solution to developing countries on high infant mortality. It will increase life expectancy for infants born prematurely where incubators are frequently sparse or rudimentary |
| 9 | | Le, H. *et al* | | 2014 | | | | USA | | | | | Indexed literature | | | | | Neonatal mortality rate | | | | Conceptual | | | | Non-disruptive innovation | | | | Product | | "Neonatal heartbeat annunciator" is a device that uses electrodes to pick up the Electrocardiogram (ECG) signal from the infant’s chest. Following electronic processing, the heartbeat is indicated as a flash of light emitting diode (LED) light and the sound of a high-frequency buzzer | Academy AND Policy makers | Neonatal heartbeat annunciator is quickly able to detect heartbeats from the ECG simulator in the laboratory. The device is small, low power, and easy to attach. It has met most design specifications |
| 10 | | Tran, K. *et al* | | 2014 | | | | USA | | | | | Indexed literature | | | | | Under-five mortality rate; Neonatal mortality rate | | | | Empirical, laboratory research | | | | Non-disruptive innovation | | | | Product | | A thermodynamically advanced low-cost incubator that features three innovations: a disposable baby chamber, a passive cooling mechanism using low-cost heat pipes and evaporative cooling from locally found clay pots, and insulated panels and a thermal bank consisting of water that effectively preserve and store heat | Academy | The incubator design was originally intended for hospital use only, but after surveying end target users, it has been determined that the incubator can be extended to home use because of its low maintenance |
| 11 | Lund, S. *et al* | | 2014 | | | | Denmark | | | | | Indexed literature | | | | | Neonatal mortality rate | | | | Empirical, experimental research | | | | Non-disruptive innovation | | | | Process | | | The "Wired Mothers" mobile phone intervention was designed aiming to link pregnant women to their primary health care provider throughout their pregnancy, childbirth, and postpartum period | Academy AND Policy makers | The initiative was associated with a reduction in perinatal mortality; The overall perinatal mortality rate in the study was 27 per 1000 total births. The rate was lower in the intervention clusters, 19 per 1000 births, than in the control clusters, 36 per 1000 births. The intervention was associated with a significant reduction in perinatal mortality with an odds ratio (OR) of 0.50 (95% CI 0.27-0.93). Other secondary outcomes showed an insignificant reduction in stillbirth (OR 0.65, 95% CI 0.34-1.24) and an insignificant reduction in death within the first 42 days of life (OR 0.79, 95% CI 0.36-1.74) |
| 12 | Turab, *A. et al* | | 2014 | | | | Canada | | | | | Indexed literature | | | | | Neonatal mortality rate | | | | Empirical, experimental research | | | | Non-disruptive innovation | | | | Product | | | A delivery program of a portable, low-cost, integrated neonatal kit comprised of evidence-based interventions to pregnant women during their third trimester of pregnancy and the provision of education on how to use the individual kit components | Academy AND Policy makers | No data available (ongoing) |
| 13 | Jo, Y. *et al* | | 2014 | | | | USA | | | | | Indexed literature | | | | | Under-five mortality rate; Neonatal mortality rate | | | | Conceptual | | | | Non-disruptive innovation | | | | Structural | | | "Lives Saved Tool (LiST)" is an evidence based modelling software to identify priority areas for maternal and neonatal health services, by formulating six individual and combined interventions scenarios | Academy AND Policy makers | For the bundled interventions, all-combined interventions at 50% coverage scenario demonstrated the largest impact in terms of neonatal mortality; findings show that skilled birth attendance and increased facility delivery as targets for mobile Health strategies are likely to provide the biggest mortality impact relative to other intervention scenarios |
| 14 | Kumutha, J. *et al* | | 2014 | | | | India | | | | | Indexed literature | | | | | Neonatal mortality rate | | | | Empirical, longitudinal research | | | | Disruptive innovation | | | | Structural | | | The National Rural Health Mission (NRHM) is a program addressing the health needs of underserved rural areas, establishing a fully functional, community owned, decentralized health delivery system, to ensure simultaneous action on a wide range of determinants of health such as water, sanitation, education, nutrition, social and gender equality | Policy makers | Tamil Nadu has reached its Millennium Development Goals and is ensuring sustained progress in reducing child and maternal mortality with an effective implementation of the various schemes of NRHM |
| 15 | | M-Chanjo | | 2014 | | | | Kenya | | | | | Non-indexed literature | | | | | | Under-five mortality rate | | | | Empirical, longitudinal research | | | | Non-disruptive innovation | | | | Process | "M-Chanjo" is an app that creates awareness on child immunization among parents by sending text message reminders | Companies | M-Chanjo success as an mobile Health platform that disseminates information on childhood vaccines is due to the wide availability of cheap phones in the countries of Africa |
| 16 | | UNICEF | | 2014 | | | | USA | | | | | Non-indexed literature | | | | | | Under-five mortality rate | | | | Empirical, longitudinal research | | | | Non-disruptive innovation | | | | Process | "ARIDA (Acute Respiratory Infection Diagnostic Aid)" project is linked to development of handheld devices that accurately diagnose pneumonia in children. It includes a Children’s Automatic Respiratory Monitor and a joint Respiratory Rate and oximetry device | Companies AND Policy makers | After 3 years of implementation, 1.3 million children accessed treatment for pneumonia after diagnosis with new devices; 6,175 health workers trained and equipped with devices in Bolivia, Ethiopia and Nepal |
| 17 | | Concern Worldwide | | 2014 | | | | Ireland | | | | | Non-indexed literature | | | | | | Neonatal mortality rate | | | | Empirical, longitudinal research | | | | Non-disruptive innovation | | | | Structural | "Essential Newborn Care Corps" is a program where traditional birth attendants, instead of delivering babies, visit pregnant mothers and newborns at home, check for danger signs and refer them to the health centre for complications, antenatal and postnatal checks, and deliveries. Besides, they teach the parents about health and nutrition, a delivery plan, and methods of family planning. At the same time, they earn money by selling health products the women need | Academy AND Companies AND Policy makers | Since the program was launched, Bo District health centres have received thousands of visits from women and newborns referred by the health promoters |
| 18 | | Kim, S. | | 2015 | | | | Korea | | | | | Indexed literature | | | | | | Under-five mortality rate | | | | Conceptual | | | | Non-disruptive innovation | | | | Process | An Infant Health Condition Check Solution, composed by a wearable device and a camera, capable of attitude measuring and recognition functionalities based on the Attitude Heading Reference System and image recognition system | Academy AND Companies |  |
|  |  |  |  |  |  |  |  |  |  |  |  |  |  |  |  |  |  |  |  |  |  |  |  |  |  |  |  |  |  |  |  |  |  | This innovation coped with some emergency situations through the real-time data transmission, and it can get a very high accuracy about the infant's attitude via image recognition |
| 19 | | Ghana Health Services; University of Ghana | | 2015 | | | | Ghana | | | | | Non-indexed literature | | | | | | Neonatal mortality rate | | | | Empirical, experimental research | | | | Non-disruptive innovation | | | | Process | The program´s approach resides on text messaging of standard protocols for maternal and neonatal care to front line health care providers. These contents will support clinical decision making by frontline health care professionals on neonatal and maternal mortality | Academy AND Policy makers | No data available |
| 20 | | Adams, V. *et al* | | 2016 | | | | USA | | | | | Indexed literature | | | | | | Under-five mortality rate | | | | Empirical, longitudinal research | | | | Disruptive innovation | | | | Structural | "One heart world-wide (OHW): The network of safety model" is an approach that, focusing on one community or catchment area at a time, tries to build a complete network in a small area, instead of trying to scale up quickly or provide a single, vertical intervention across a vast region or population | Policy makers | The results of this program approach have so far been positive |
| 21 | | Awiti, A.M. *et al* | | | 2016 | | | | Uganda | | | | | Indexed literature | | | | | Neonatal mortality rate | | | | Empirical, focus group | | | | Non-disruptive innovation | | | | Process | Android Based Digital Fetoscope is an application created to facilitate fetal heart monitoring and potentially reduce the number of neonatal deaths | Academy AND Policy makers | The work presents a novel and feasible option for affordable digital fetal heart rate monitoring in low- and medium-income countries |
| 22 | | Kumaresh, S. *et al* | | | 2016 | | | | India | | | | | Indexed literature | | | | | Under-five mortality rate | | | | Empirical, focus group | | | | Non-disruptive innovation | | | | Process | An Android mobile application capable of non-invasive fetal heart rate and growth measurement with abnormality detection using the Internet of Things concept | Academy | By using this approach, the mother’s and fetal heart rates are measured from week 5 to week 40 of pregnancy |
| 23 | | Ferreira, A. G. *et al* | | | 2016 | | | | Portugal | | | | | Indexed literature | | | | | Neonatal mortality rate | | | | Empirical, laboratory research | | | | Non-disruptive innovation | | | | Process | The "Baby Night Watch" project consists of a Smart Wearable System composed by a Wearable Internet of Things device, a Gateway and a medical interface | Academy | The "Baby Night Watch" is capable of detecting unexpected events and registering several physiological parameters; The project proved that with a small amount of hardware a huge number of parameters can be measured |
| 24 | | Concern Worldwide | | | 2016 | | | | Ireland | | | | | Non-indexed literature | | | | | Under-five mortality rate | | | | Empirical, longitudinal research | | | | Non-disruptive innovation | | | | Structural | "PlanWise" is a program that helps planners and responders in low-resource settings see how they can serve as many people as they can, as cost-effectively as possible, to help saving the lives of mothers and babies. It is based on an algorithm that draws on freely available public data | Academy AND Companies AND Policy makers | Tests showed that the mathematical model of the algorithm was accurate |
| 25 | | dela Cruz, D.R. *et al* | | | 2017 | | | | Philippines | | | | | Indexed literature | | | | | Under-five mortality rate | | | | Empirical, survey (n=32) | | | | Non-disruptive innovation | | | | Process | Milktrack is a mobile application that will help reducing infant mortality rate and preventing undernutrition and stunted growth among children through empowering the breastfeeding practice | Academy | This mobile application serves as an effective way of promoting and enhancing breastfeeding practice in the Philippines |
| 26 | | Ghandour, R.M. *et al* | | | 2017 | | | | USA | | | | | Indexed literature | | | | | Under-five mortality rate | | | | Empirical, longitudinal research | | | | Disruptive innovation | | | | Structural | "The Collaborative Improvement & Innovation Network (CoIIN)" model is an innovative approach, using the science of quality improvement and collaborative learning to bolster existing policy, clinical and system-level efforts and develop innovative approaches to accelerate improvement in birth outcomes. | Policy makers | It offers a promising approach to strengthening partnerships within and across states, bolstering data systems to inform and track progress more rapidly, accelerating improvement toward healthier communities, States, and the Nation as a whole. |
| 27 | | Vora, S.A. *et al* | | | 2017 | | | | USA | | | | | Indexed literature | | | | | Under-five mortality rate | | | | Empirical, laboratory research | | | | Non-disruptive innovation | | | | Product | A seamless wearable infant monitor - it is an unconventional battery-free and wireless infant heart and respiration rate monitor that uses passive Radio-Frequency Identification (RFID) technology | Academy | Bradycardia and apnea detection are possible by simultaneously using novel RFID based heart and respiration rate monitors. The heart rate monitor displays a correlation of over 99% with the actual heart rate even in the presence of the respiration rate tag. The respiration monitor is able to detect an apnea within 10 seconds of its onset |
| 28 | | Amirneni, S. *et al* | | | 2017 | | | | India | | | | | Indexed literature | | | | | Under-five mortality rate | | | | Conceptual | | | | | Non-disruptive innovation | | | Process | A cloud-based system that tracks the pregnant woman right from the early pregnancy stage till the child turns the age of five | Academy AND Policy makers | The intensive tracking provided by this system at both prenatal and postnatal conditions will contribute to the decrease in child mortality rate in rural areas |
| 29 | | Esamai, F. *et al* | | | 2017 | | | | Kenya | | | | | Indexed literature | | | | | Under-five mortality rate; Neonatal mortality rate | | | | Empirical, longitudinal research | | | | | Non-disruptive innovation | | | Process | "Enhanced Health Care System (EHC)" is a program that embodies six WHO pillars of the health system and community owned initiatives. It is an innovative system approach on maternal, neonatal and under-five children’s outcomes | Academy AND Policy makers | The expected findings from the study include showing trends in improvement in the intervention clusters for morbidity, mortality, health service utilization and access indicators |
| 30 | | Amani, A. *et al* | | | 2017 | | | | Cameroon | | | | | Indexed literature | | | | | Under-five mortality rate | | | | Empirical, longitudinal research | | | | | Non-disruptive innovation | | | Process | “Yaoundé Perinatal Network" is a perinatal transfer platform based on WhatsApp messenger application. It allows a twice-daily updated status of the available equipment (incubators, oxygen and phototherapy) and bed capacities across paediatric units, leading to quick interaction between the network members and an immediately preparation and baby transfer | Academy AND Policy makers | Even though there are not baseline data for comparisons, preliminary results are somehow indicative of promising changes towards early neonatal care |
| 31 | | da Silva, C.L. *et al* | | | 2017 | | | | Brazil | | | | | Indexed literature | | | | | Under-five mortality rate | | | | Empirical, laboratory research | | | | | Non-disruptive innovation | | | Process | "LAIS" (Intelligent  Health System Analyzer) is an intelligent mechanism that uses machine learning to generate child death risk alerts in GISSA (Intelligent Governance Framework  for Brazilian Health System) .GISSA is an intelligent system for health decision making focused on children maternal care) | Academy AND Policy makers | The tests show that the Naive Bayes classifier is the most suitable algorithm for this purpose, presenting good results with a ROC curve of 92.1% |
| 32 | | Vigliotti, V.S. *et al* | | | 2018 | | | | USA | | | | | Indexed literature | | | | | Under-five mortality rate | | | | Empirical, laboratory research | | | | | Disruptive innovation | | | Process | "Clustered regularly interspaced short palindromic repeats (CRISPR)" are public health innovations related to gene-editing technologies | Academy | CRISPR/Cas9 is a tool that can help save lives, from matters such as eradicating malaria to influenza pandemics and even to the shortage of available organ donations |
| 33 | | Charpak, N. *et al* | | | 2018 | | | | Columbia | | | | | Indexed literature | | | | | Under-five mortality rate; Neonatal mortality rate | | | | Empirical, analysis of documents | | | | | Disruptive innovation | | | Structural | "Kangaroo Mother Care (KMC)" method is the oldest innovation currently available to reduce neonatal and infant mortality. It includes three major innovative approaches: kangaroo position, kangaroo nutrition and kangaroo discharge policies | Academy AND Policy makers | KMC is now an evidenced-based method in all its aspects |
| 34 | | Mothi, M.S. *et al* | | | 2018 | | | | Malaysia | | | | | Indexed literature | | | | | Under-five mortality rate | | | | Empirical, laboratory research | | | | | Non-disruptive innovation | | | Product | A continuous infant temperature alerting system that, in case of temperature crossing threshold value, produces a warning message alerting the parent. The location of the infant is found and an instant alert message is sent to the doctor/Village Health Officer | Academy | The infant temperature measuring device allows the illiterate peoples in rural areas to easily identify the problem in baby with help of sound and light for different temperature values like mild fever, normal fever and heavy fever |
| 35 | | O'Connor, Y. *et al* | | | | 2018 | | | | Ireland | | | | | Indexed literature | | | | Under-five mortality rate | | | | Empirical, experimental research | | | | | Non-disruptive innovation | | | Process | "Supporting LIFEeCCM App (Supporting Low-cost Intervention For disEase control and Community Case Management App)" is a mobile Health intervention whose hardware, software, and system configurations interact with the sociocultural and economic context, supporting clinical decisions | Academy AND Policy makers | The app works effectively on the ground supporting healthcare workers to do their jobs in conducting health assessments of young children in the community. Concrete data are not available |
| 36 | | Shift Labs, Inc. | | | | 2018 | | | | USA | | | | | Non-indexed literature | | | | Neonatal mortality rate | | | | Conceptual | | | | | Non-disruptive innovation | | | Process | DripAssist Infusion Rate Monitor is an Intravenous (IV) infusion technology to reduce maternal and newborn deaths from conditions such as postpartum haemorrhage and eclampsia, allowing clinicians to easily manage IV infusions without the cost or complexity of traditional infusion pumps | Companies | No data available |
| 37 | | Tipene-Leach, D. *et al* | | | | 2019 | | | | New Zealand | | | | | Indexed literature | | | | Under-five mortality rate; Neonatal mortality rate | | | | Empirical, analysis of documents | | | | | Non-disruptive innovation | | | Product | The bassinet-like wahakura is an in-bed safe sleep device made of native flax that would disrupt the Sudden Unexpected Infant Death risk associated with ´bedsharing where there was smoking in pregnancy’ without counting on smoking cessation | Academy AND Policy makers | It was found solid evidence that the intervention was appropriate, safe and effective |
| 38 | | Gueye, K. *et al* | | | | 2019 | | | | Senegal | | | | | Indexed literature | | | | Under-five mortality rate | | | | Empirical, focus group | | | | | Non-disruptive innovation | | | Process | An Health Care System platform based on the Web Real-Time Communication (WebRTC) Kurento multimedia server and the Web of Things (WoT). It is a telemedicine solution to help rural populations access quality health care services | Academy | This system significantly reduces expenses and increases the quality of life of patients. it will be possible for a mother to be consulted by the best specialists of the country without leaving her locality |
| 39 | | Moreira, M.W.L. *et al* | | | | 2019 | | | | Brazil | | | | | Indexed literature | | | | Under-five mortality rate; Neonatal mortality rate | | | | Empirical, laboratory research | | | | | Non-disruptive innovation | | | Process | This paper proposes an analysis of several Machine Learning (ML) techniques capable of predicting whether the fetus will be born small for its gestational age, allowing directed intervention. | Academy | Hybrid methods based on ensemble learning are capable of efficiently predicting the expected weight of the fetus at birth. The "bagged tree model", an hybrid model, achieved excellent results concerning accuracy and area under the receiver operating characteristic curve, to know, 0.849 and 0.636, respectively |
| 40 | | Mantari, A.A. *et al* | | | | 2019 | | | | Peru | | | | | Indexed literature | | | | Under-five mortality rate | | | | Empirical, laboratory research | | | | | Non-disruptive innovation | | | Process | An Automatic algorithm that differentiates between normal and abnormal lung sounds | Academy | It was possible to find a regression model that correctly classified 97.44% of the sample sounds, and when categorizing the results, 100% was found in sensitivity and specificity |
| 41 | | Patterson, J.K. *et al* | | | | 2019 | | | | | USA | | | | Indexed literature | | | | Under-five mortality rate; Neonatal mortality rate | | | | | Empirical, analysis of documents | | | | Non-disruptive innovation | | | Product | Three innovations are described, coupling cardiorespiratory monitoring with Helping Babies Breathe (HBB) approach: NeoNatalie Live, the Augmented Infant Resuscitator, and NeoBeat. NeoNatalie Live is a high-fidelity manikin that facilitates bag mask ventilation training through case scenarios of varying difficulty. The Augmented Infant Resuscitator is added in-line between a face mask and ventilation bag during bag mask ventilation training to provide users with real-time feedback on ventilation quality. NeoBeat is a battery-operated heart rate meter that digitally displays the newborn heart rate during bedside resuscitations | Academy | Although more data are needed, cardiorespiratory monitoring during training as well as bedside resuscitations may improve resuscitation care and newborn outcomes in Lower-Middle-Income Countries |
| 42 | | UNICEF | | | | 2019 | | | | | USA | | | | Non-indexed literature | | | | Under-five mortality rate | | | | | Empirical, longitudinal research | | | | Non-disruptive innovation | | | Process | The "Scaling Pneumonia Response INnovaTions (SPRINT) Project" is a country-level triaging tool for the scale up of oxygen therapy and amoxicillin dispersible tablets among children. | Policy makers | The Ministries of Health in Senegal and Ghana are currently introducing SPRINT in targeted districts together with the United Nations International Children's Emergency Fund (UNICEF), with plans to use the model for continued national expansion beyond the targeted regions and districts |
| 43 | | Bill and Melinda Gates Foundation | | | | 2020 | | | | | USA | | | | Non-indexed literature | | | | Under-five mortality rate | | | | | Empirical, longitudinal research | | | | Non-disruptive innovation | | | Process | The program focuses on optimizing the speed, responsiveness and efficiency of the existing medical supply chain, through the use of drones to improves access to essential medical supplies | Policy makers | Researchers are "with a strong view that this innovation will reduce our maternal and child mortality and morbidity, improve health outcomes and strengthen our supply chain into a more resilient outfit." |
| 44 | | Armenia SDG Innovation Lab | | | | 2020 | | | | | Armenia | | | | Non-indexed literature | | | | Under-five mortality rate | | | | | Empirical, longitudinal research | | | | Non-disruptive innovation | | | Process | "Barev Balik" is a maternal mobile Health app and web portal to modernize and improve the maternal healthcare system in Armenia | Companies AND Policy makers | No data available (ongoing) |
| 45 | | Universidad Peruana Cayetano Heredia | | | | 2020 | | | | | Peru | | | | Non-indexed literature | | | | Under-five mortality rate; Neonatal mortality rate | | | | | Empirical, longitudinal research | | | | Disruptive innovation | | | Structural | "Mamás del Río" is a programme that provides training to midwives and Community Health Agents to know how to detect emergency signs of pregnancy, childbirth, postpartum and newborn, and prepares for home delivery, in case institutional delivery is not feasible. The Community Health Agents educates through tablets and educational materials and three educational booklets are provided to mothers | Academy AND Companies AND Policy makers | Following the 18-month pilot study, the percentage of women receiving prenatal care in the first trimester increased from 38 per cent to 63 per cent and institutional delivery assistance doubled from 16 per cent to 37 per cent, reducing complications and providing timely response to potential maternal and perinatal emergencies |
| 46 | | Safe Motherhood Alliance | | | | 2020 | | | | | Zambia | | | | | Non-indexed literature | | | | Under-five mortality rate; Neonatal mortality rate | | | | Empirical, longitudinal research | | | | Disruptive innovation | | | Structural | A programme where traditional birth attendants are trained to deliver improved medical products to their communities, and each is equipped with Safe Motherhood Alliance’s flagship product — "Baby Delivery Kits", which they sell to mothers. It contains personal protective equipment (including 3D printed face masks and visors) and baby delivery products (biodegradable sanitary pads, sterilizing agents, umbilical cord clips, plastic gloves, surgical blades, cotton wool, and more) | Policy makers | This innovation serves the needs of new mothers and helps to support girls in the wider community by selling kits at an affordable price |
| 47 | | Uniscientia foundation, Venture Kick, and Wyss Zurich | | | | 2020 | | | | | Switzerland | | | | | Non-indexed literature | | | | Neonatal mortality rate | | | | Empirical, laboratory research | | | | Non-disruptive innovation | | | Product | "OxyPrem" is a novel oximeter improving intensive care by avoiding oxygen undersupply and brain damage | Academy AND Companies | OxyPrem achieves new levels in precision, demonstrating excellent performance in repeated placement and high reading certainty. High component quality and advanced algorithms provide consistent readings, also across varying perfusion environments |
| 48 | | Hasan, S. *et al* | | | | 2021 | | | | | India | | | | | Indexed literature | | | | Under-five mortality rate | | | | Empirical, laboratory research | | | | Non-disruptive innovation | | | Product | An android application developed to make sure children have access to healthcare and immunization against vaccine prevented diseases. It provides a system to provide information, store records and help parents schedule vaccination appointments for their children | Academy | The application worked correctly. The described system fulfilled its main purposes |
| 49 | | UNICEF | | | | 2021 | | | | | USA | | | | | Non-indexed literature | | | | Under-five mortality rate | | | | Empirical, longitudinal research | | | | Non-disruptive innovation | | | Structural | UNICEF’s "Oxygen Therapy Project" aims to provide governments with practical tools for building oxygen systems in their countries. Its program includes an oxygen system planning tool, an interagency technical specifications and guidance manual and a supply Catalogue | Policy makers | No data available (currently being introduced and tested) |
| 50 | | UNICEF | | | | 2021 | | | | | USA | | | | | Non-indexed literature | | | | Under-five mortality rate | | | | Conceptual | | | | Non-disruptive innovation | | | Process | The innovative Vaccine MicroArray Patches (VMAPs) provide an intradermal delivery technology for vaccinations | Policy makers | VMAPs can directly contribute to increasing immunization coverage and decreasing the occurrence and impact of outbreaks |
| 51 | | University of California, San Francisco | | | | 2021 | | | | | USA | | | | | Non-indexed literature | | | | Under-five mortality rate | | | | Empirical, experimental research | | | | Non-disruptive innovation | | | Process | Investigators hypothesize that biannual administration of azithromycin to children aged 1-11 months will reduce mortality in this age group, aiming to demonstrate that this intervention can be scaled up and produce the same benefits on mortality as those documented in smaller, more controlled studies | Academy | No data available (not yet recruiting, at the time) |
