# Supplementary material for: Characterization of Innovation to Fight Child Mortality: A Systematic Scoping Review
Source: Int J Public Health. 2022 Aug 15;67:1604815. doi: 10.3389/ijph.2022.1604815 (PMC9421644; doi:10.3389/ijph.2022.1604815)
Supplement: Supplementary file 2 [file DataSheet1.docx]

**IJPH - Supplementary material - Characterization of Innovation to fight Child Mortality, A Systematic Scope Review**

**Supplementary Material 1 (Indexed Literature Search Expressions)**

**Pubmed/MEDLINE**

("child mortality"[Mesh] OR "child mortality"[Title/Abstract] OR "child death"[Title/Abstract] OR "childbirth complications"[Title/Abstract] OR "neonatal mortality"[Title/Abstract] OR "neonatal death"[Title/Abstract] OR "neo-natal mortality"[Title/Abstract] OR "neo-natal death"[Title/Abstract] OR "premature mortality"[Title/Abstract] OR "premature death"[Title/Abstract] OR "infant mortality"[Title/Abstract] OR "infant death"[Title/Abstract] OR "perinatal mortality"[Title/Abstract] OR "perinatal death"[Title/Abstract] OR "under 5 mortality"[Title/Abstract] OR "under 5 death"[Title/Abstract] OR "under five mortality"[Title/Abstract] OR "under five death"[Title/Abstract] ) AND ("organizational innovation"[Mesh] OR "innovation"[Title/Abstract] OR “creativ*”[Title/Abstract] OR “invent*”[Title/Abstract] OR “triple helix model"[Title/Abstract] OR "quadruple helix model"[Title/Abstract] OR "innovation cooperation"[Title/Abstract] OR "innovation partnership*"[Title/Abstract] OR "innovation approach"[Title/Abstract] OR "innovation process"[Title/Abstract] OR "innovation project"[Title/Abstract] OR "service* innovation"[Title/Abstract] OR "innovation adoption"[Title/Abstract] OR "personalized innovation"[Title/Abstract] OR "diffusion of innovation"[Title/Abstract] OR "digital innovation"[Title/Abstract] OR "implementation of innovation"[Title/Abstract] OR "technological innovation"[Title/Abstract] OR "entrepreneur*"[Title/Abstract])

**Web of Science**

(TI=("child mortality" OR "child death" OR "childbirth complications" OR "neonatal mortality" OR "neonatal death" OR "neo*natal mortality" OR "neo*natal death" OR "premature mortality" OR "premature death" OR "infant mortality" OR "infant death" OR "peri*natal mortality" OR "peri*natal death" OR "under*5 mortality" OR "under*5 death" OR "under*five mortality" OR "under*five death" ) OR AB=("child mortality" OR "child death" OR "childbirth complications" OR "neonatal mortality" OR "neonatal death" OR "neo*natal mortality" OR "neo*natal death" OR "premature mortality" OR "premature death" OR "infant mortality" OR "infant death" OR "peri*natal mortality" OR "peri*natal death" OR "under*5 mortality" OR "under*5 death" OR "under*five mortality" OR "under*five death" ) OR KP=("child mortality" OR "child death" OR "childbirth complications" OR "neonatal mortality" OR "neonatal death" OR "neo*natal mortality" OR "neo*natal death" OR "premature mortality" OR "premature death" OR "infant mortality" OR "infant death" OR "peri*natal mortality" OR "peri*natal death" OR "under*5 mortality" OR "under*5 death" OR "under*five mortality" OR "under*five death" )) AND (TI=("innovation" OR “creativ*” OR “invent*” OR "triple helix model" OR "quadruple helix model" OR "innovation cooperation" OR "innovation partnership*" OR "innovation approach" OR "innovation process" OR "innovation project" OR "service* innovation" OR "innovation adoption" OR "personalized innovation" OR "diffusion of innovation" OR "digital innovation" OR "implementation of innovation" OR "technological innovation" OR "entrepreneur*") OR AB=("innovation" OR “creativ*” OR “invent*” OR "triple helix model" OR "quadruple helix model" OR "innovation cooperation" OR "innovation partnership*" OR "innovation approach" OR "innovation process" OR "innovation project" OR "service* innovation" OR "innovation adoption" OR "personalized innovation" OR "diffusion of innovation" OR "digital innovation" OR "implementation of innovation" OR "technological innovation" OR "entrepreneur*") OR KP=("innovation" OR “creativ*” OR “invent*” OR "triple helix model" OR "quadruple helix model" OR "innovation cooperation" OR "innovation partnership*" OR "innovation approach" OR "innovation process" OR "innovation project" OR "service* innovation" OR "innovation adoption" OR "personalized innovation" OR "diffusion of innovation" OR "digital innovation" OR "implementation of innovation" OR "technological innovation" OR "entrepreneur*"))

**Scopus**

TITLE-ABS-KEY ( ( "child mortality" OR "child death" OR "childbirth complications" OR "neonatal mortality" OR "neonatal death" OR "neo*natal mortality" OR "neo*natal death" OR "premature mortality" OR "premature death" OR "infant mortality" OR "infant death" OR "peri*natal mortality" OR "peri*natal death" OR "under*5 mortality" OR "under*5 death" OR "under*five mortality" OR "under*five death" ) AND ( "innovation" OR “creativ*” OR “invent*” OR "triple helix model" OR "quadruple helix model" OR "innovation cooperation" OR "innovation partnership*" OR "innovation approach" OR "innovation process" OR "innovation project" OR "service* innovation" OR "innovation adoption" OR "personalized innovation" OR "diffusion of innovation" OR "digital innovation" OR "implementation of innovation" OR "technological innovation" OR "entrepreneur*"))

**IEEEXplore**

("Document Title":"child mortality" OR "Document Title":"child death" OR "Document Title":"childbirth complications" OR "Document Title":"neonatal mortality" OR "Document Title":"neonatal death" OR "Document Title":"neo*natal mortality" OR "Document Title":"neo*natal death" OR "Document Title":"premature mortality" OR "Document Title":"premature death" OR "Document Title":"infant mortality" OR "Document Title":"infant death" OR "Document Title":"peri*natal mortality" OR "Document Title":"peri*natal death" OR "Document Title":"under*5 mortality" OR "Document Title":"under 5 death" OR "Document Title":"under five mortality" OR "Document Title":"under five death") AND ("Document Title":"innovation" OR "Document Title":“creativ*” OR "Document Title":“invent*” OR "Document Title":"triple helix model" OR "Document Title":"quadruple helix model" OR "Document Title":"innovation cooperation" OR "Document Title":"innovation partnership*" OR "Document Title":"innovation approach" OR "Document Title":"innovation process" OR "Document Title":"innovation project" OR "Document Title":"service* innovation" OR "Document Title":"innovation adoption" OR "Document Title":"personalized innovation" OR "Document Title":"diffusion of innovation" OR "Document Title":"digital innovation" OR "Document Title":"implementation of innovation" OR "Document Title":"technological innovation" OR "Document Title":"entrepreneur*") OR ("Abstract":"child mortality" OR "Abstract":"child death" OR "Abstract":"childbirth complications" OR "Abstract":"neonatal mortality" OR "Abstract":"neonatal death" OR "Abstract":"neo*natal mortality" OR "Abstract":"neo*natal death" OR "Abstract":"premature mortality" OR "Abstract":"premature death" OR "Abstract":"infant mortality" OR "Abstract":"infant death" OR "Abstract":"peri*natal mortality" OR "Abstract":"peri*natal death" OR "Abstract":"under*5 mortality" OR "Abstract":"under 5 death" OR "Abstract":"under five mortality" OR "Abstract":"under five death") AND ("Abstract":"innovation" OR "Abstract":“creativ*” OR "Abstract":“invent*” OR "Abstract":"triple helix model" OR "Abstract":"quadruple helix model" OR "Abstract":"innovation cooperation" OR "Abstract":"innovation partnership*" OR "Abstract":"innovation approach" OR "Abstract":"innovation process" OR "Abstract":"innovation project" OR "Abstract":"service* innovation" OR "Abstract":"innovation adoption" OR "Abstract":"personalized innovation" OR "Abstract":"diffusion of innovation" OR "Abstract":"digital innovation" OR "Abstract":"implementation of innovation" OR "Abstract":"technological innovation" OR "Abstract":"entrepreneur*")

**Academic Search Ultimate**

TI("child mortality" OR "child death" OR "childbirth complications" OR "neonatal mortality" OR "neonatal death" OR "neo*natal mortality" OR "neo*natal death" OR "premature mortality" OR "premature death" OR "infant mortality" OR "infant death" OR "peri*natal mortality" OR "peri*natal death" OR "under*5 mortality" OR "under*5 death" OR "under*five mortality" OR "under*five death" ) AND ("innovation" OR “creativ*” OR “invent*” OR "triple helix model" OR "quadruple helix model" OR "innovation cooperation" OR "innovation partnership*" OR "innovation approach" OR "innovation process" OR "innovation project" OR "service* innovation" OR "innovation adoption" OR "personalized innovation" OR "diffusion of innovation" OR "digital innovation" OR "implementation of innovation" OR "technological innovation" OR "entrepreneur*") OR AB("child mortality" OR "child death" OR "childbirth complications" OR "neonatal mortality" OR "neonatal death" OR "neo*natal mortality" OR "neo*natal death" OR "premature mortality" OR "premature death" OR "infant mortality" OR "infant death" OR "peri*natal mortality" OR "peri*natal death" OR "under*5 mortality" OR "under*5 death" OR "under*five mortality" OR "under*five death" ) AND ("innovation" OR “creativ*” OR “invent*” OR "triple helix model" OR "quadruple helix model" OR "innovation cooperation" OR "innovation partnership*" OR "innovation approach" OR "innovation process" OR "innovation project" OR "service* innovation" OR "innovation adoption" OR "personalized innovation" OR "diffusion of innovation" OR "digital innovation" OR "implementation of innovation" OR "technological innovation" OR "entrepreneur*")

**Business Source Ultimate**

TI("child mortality" OR "child death" OR "childbirth complications" OR "neonatal mortality" OR "neonatal death" OR "neo*natal mortality" OR "neo*natal death" OR "premature mortality" OR "premature death" OR "infant mortality" OR "infant death" OR "peri*natal mortality" OR "peri*natal death" OR "under*5 mortality" OR "under*5 death" OR "under*five mortality" OR "under*five death" ) AND ("innovation" OR “creativ*” OR “invent*” OR "triple helix model" OR "quadruple helix model" OR "innovation cooperation" OR "innovation partnership*" OR "innovation approach" OR "innovation process" OR "innovation project" OR "service* innovation" OR "innovation adoption" OR "personalized innovation" OR "diffusion of innovation" OR "digital innovation" OR "implementation of innovation" OR "technological innovation" OR "entrepreneur*") OR AB("child mortality" OR "child death" OR "childbirth complications" OR "neonatal mortality" OR "neonatal death" OR "neo*natal mortality" OR "neo*natal death" OR "premature mortality" OR "premature death" OR "infant mortality" OR "infant death" OR "peri*natal mortality" OR "peri*natal death" OR "under*5 mortality" OR "under*5 death" OR "under*five mortality" OR "under*five death") AND ("innovation" OR “creativ*” OR “invent*” OR "triple helix model" OR "quadruple helix model" OR "innovation cooperation" OR "innovation partnership*" OR "innovation approach" OR "innovation process" OR "innovation project" OR "service* innovation" OR "innovation adoption" OR "personalized innovation" OR "diffusion of innovation" OR "digital innovation" OR "implementation of innovation" OR "technological innovation" OR "entrepreneur*")

**Supplementary Material 2 (Grey literature Search)**

| **General sources of grey literature** | |
| --- | --- |
| **Source** | **Web link** |
| OAIster | <http://oaister.worldcat.org/> |
| OpenGrey | <http://www.opengrey.eu/> |
| Global Health Observatory (GHO) Data | <https://www.who.int/data/gho> |
| The Grey Literature Report | <http://www.greylit.org/> |
| PQDT Open | <http://pqdtopen.proquest.com/> |
| OpenThesis | https://oatd.org/ |
| Thesis Commons | <http://thesiscommons.org/> |
| Carrot2 | <https://search.carrot2.org/#/search/web> |
| Millionshort | <https://millionshort.com/> |
| Google Search | [https://www.google.com](https://www.google.com/) |

| **Specific sources of grey literature** | |
| --- | --- |
| **Source** | **Web link** |
| WHO World Health Report | <https://www.who.int/whr/en/> |
| UNDP Human Development Reports | <http://hdr.undp.org/> |
| IMF World Economic Outlook Reports | <https://www.imf.org/en/publications/weo> |
| World Bank World Development Report | <https://www.worldbank.org/en/publication/wdr/wdr-archive> |
| World Bank World Development Indicators | <https://datacatalog.worldbank.org/dataset/world-development-indicators> |
| UNSD Social Indicators | <https://unstats.un.org/unsd/demographic/products/socind/default.htm> |
| ADB Asian Development Outlook | <https://www.adb.org/publications/series/asian-development-outlook> |
| ADB Key Indicators for Asia and the Pacific | <https://www.adb.org/publications/series/key-indicators-for-asia-and-the-pacific> |
| ADBG African Development Report | <https://www.afdb.org/en/documents/publications/african-development-report> |
| World Bank Africa Development Indicators | <https://datacatalog.worldbank.org/dataset/africa-development-indicators> |
| OECD Health Data | <https://library.queensu.ca/search/database/oecd-health-data> |
| WHO Global Health Observatory data | <https://www.who.int/data/gho> |
| WHO Bulletins | <https://www.who.int/bulletin/en/> |
| Other Country Information | <https://guides.library.queensu.ca/gov/foreign-international/other-countries> |
| PAHO Digital Library | <https://www.paho.org/en/paho-digital-library> |
| PAHO Health Information Platform | <https://www3.paho.org/data/index.php/en/> |
| OECD i Library | <https://www.oecd-ilibrary.org/?_ga=2.196669804.1391051612.1629762131-193869305.1628926977> |
| OECD Health Statistics 2021 | <https://www.oecd.org/health/health-data.htm> |
| UNICEF | <https://www.unicef.org/search?force=0&query=%22child+mortality%22+AND+%22innovation%22&combined_sort=relevance_desc&search_date_range_picker=&created%5Bmin%5D=&created%5Bmax%5D=&name=&name=&name=> |
| UNICEF | <https://www.unicef.org/innovation/stories/maternal-and-newborn-health-innovations-project> |
| Concern Worldwide US | <https://www.concernusa.org/project-profile/innovations-maternal-newborn-child-health/> |
| Save the children | <https://www.savethechildren.org/us/about-us/why-save-the-children> |
| Bill and Melinda Gates Foundation | <https://www.gatesfoundation.org/our-work/programs/global-development/maternal-newborn-and-child-health%5C> |
| PATH | <https://www.path.org/articles/innovations-to-end-preventable-deaths-of-mothers-and-children/> |
| Healthy Newborn Network | <https://www.healthynewbornnetwork.org/blog/innovations-in-maternal-newborn-and-child-health/> |
| NCSL | <https://www.ncsl.org/Portals/1/Documents/Health/Infant-Maternal-Mortality_v05_web.pdf> |
| HRSA | <https://mchb.hrsa.gov/maternal-child-health-initiatives/collaborative-improvement-innovation-networks-coiins> |
| USAID | <https://www.usaid.gov/madagascar/ending-preventable-child-and-maternal-deaths> |
| Every woman Every child | <http://www.everywomaneverychild.org/images/Report3Businesses.pdf> |
| Centre for health market and innovations | <https://healthmarketinnovations.org/health-focus/maternal-newborn-and-child-health> |
| BIRAC | <https://www.birac.nic.in/news_description.php?id=133> |
| AMHCP | <http://www.amchp.org/programsandtopics/womens-health/Focus%20Areas/infantmortality/Documents/amchpbestpractices.pdf> |
| UN report | <https://www.who.int/pmnch/topics/maternal/201006_jap_pamphlet/en/> |
| GBD SDG | <https://vizhub.healthdata.org/child-mortality> |

| **Clinical trials registry platforms** | |
| --- | --- |
| Source | **Web link** |
| ClinicalTrials.gov | <https://clinicaltrials.gov/> |
| Clinical Trials Registries by Country | <https://en.wikipedia.org/wiki/Clinical_trials_registry#Clinical_trial_registries_by_country> |
| European Union Clinical Trials Register | <https://www.clinicaltrialsregister.eu/ctr-search/search;jsessionid=69jQXdUpjKQicm2LqmJQM97z5xW9ewGYLQvMO_V3Mj_Nz3oWT-IX!-774786170> |
| International Clinical Trials Registry Platform (WHO) | <http://apps.who.int/trialsearch/> |
| ISRCTN Registry | <http://www.isrctn.com/> |
| OpenTrials | <http://explorer.opentrials.net/> |
